# Abuse liability of two electronic nicotine delivery systems compared with combustible cigarettes and nicotine gum from an open-label randomized crossover study

**DOI:** 10.1038/s41598-023-45894-7

**Published:** 2023-11-02

**Authors:** Chris Campbell, Tao Jin, Elaine K. Round, Paul R. Nelson, Sarah Baxter

**Affiliations:** 1 Winston‑Salem, USA; 2 RAI Services Company, 401 N. Main Street, Winston‑Salem, NC 27101 USA; 3 British American Tobacco (Investments) Limited, Research and Development, Regents Park Road, Southampton, SO15 8TL UK

**Keywords:** Clinical pharmacology, Biomarkers, Medical research

## Abstract

An assessment of the likelihood of use and abuse potential for new tobacco products is an important part of tobacco product regulation in the United States and abroad. This paper reports the results of a randomized, open-label, crossover clinical study that assessed factors related to product adoption and abuse liability (AL), comparing two closed electronic nicotine delivery system (ENDS) products to combustible cigarettes and nicotine gum, high- and low-AL comparator products, respectively. During an 11-day confinement period that included multiple product familiarization sessions, healthy adult smokers participated in AL test sessions to evaluate the abuse liability of each product. During these test sessions, changes in subjective measures; speed and amount of nicotine uptake; and maximum changes in physiological effects before, during, and after use of each assigned product were assessed over 4 h. Positive subjective effects measures scores such as product-liking and overall intent to use again were highest for cigarettes, followed by the Vuse ENDS, with nicotine gum consistently having the lowest scores. The PK results (C_max_ and T_max_) of the Vuse ENDS products are between UB cigarettes and nicotine gum, which correlates with the subjective effects. All nicotine uptake measures for the Vuse ENDS products were lower than that of usual brand (UB) cigarettes, including peak nicotine uptake and overall nicotine uptake, and were either similar to or lower than nicotine gum. The time course of nicotine uptake after use of the ENDS was more similar to that of combustible cigarettes than nicotine gum. The results indicate that the AL of each ENDS product is lower than that of UB cigarettes and similar to that of nicotine gum.

## Introduction

Electronic nicotine delivery systems (ENDS), which heat a solution containing nicotine and typically propylene glycol and glycerin without causing combustion, generate aerosol containing substantially fewer and lower levels of toxicants relative to tobacco cigarette smoke^1–3^. Users of ENDS are exposed to less harmful chemical constituents than smokers of cigarettes, placing ENDS on the lower end of the risk continuum of nicotine-containing products^4–7^. Short-term switching studies (5 days to 6 weeks) have demonstrated that some smokers can effectively maintain nicotine consumption while reducing toxicant exposure, suggesting ENDS use could provide public health benefits to smokers^8–13^.

The continued growth of ENDS use and the multitude of available brands, styles, and flavors has resulted in concerns about the products’ potential abuse liability (AL)^14,15^. AL is the likelihood of engaging in persistent and problematic use of a substance that will lead to undesirable consequences, including dependence^16^. The abuse liability of an ENDS product is driven not only by the speed, amount, and manner of nicotine delivery but also by subjective factors such as positive and negative effects and product likability^17–19^. Although there are concerns about the AL of ENDS products, including the potential for nicotine delivery to be similar to that of combusted cigarettes, the same nicotine and non-nicotine factors that drive ENDS AL are also related to the success of lower-risk products such as ENDS in supplanting cigarette use among smokers^20^.

Multiple methodologies have been employed to assess a product’s AL in humans, most of which involve comparing the product of interest to comparators with known levels of abuse liability. One such methodology is based on a standard approach for pharmaceutical AL assessment that has been adapted for tobacco products^16,21,22^. Using variations of this approach, the AL of several commercially available ENDS products have been assessed and compared against combustible cigarettes (known to have high AL), nicotine replacement therapies (known to have low AL), and other products in the same tobacco categories^23–29^. In the current study, we evaluated elements of abuse liability, including nicotine pharmacokinetics and subjective measures, for Vuse Vibe Original (3.0% nicotine) and Vuse Ciro Original (1.5% nicotine) relative to combustible cigarettes and nicotine gum^30–32^. These data formed part of the premarket tobacco product applications submitted to the FDA Center for Tobacco Products, which supported FDA’s issuance of marketing granted orders for both products in 2022^33^.

## Methods

### Study design and participants

This study was designed as a randomized, open-label, crossover trial conducted in a confinement setting to independently assess the abuse liability of three commercially available ENDS products, compared to subjects’ usual brand (UB) combustible cigarettes and nicotine gum (ClinicalTrials.gov identifier: NCT03126357). The results on one of the product, Vuse Solo G2, were published previously along with detailed information on study methods and procedures^34^. The protocol and all relevant materials were reviewed and approved by Chesapeake Institutional Review Board (Columbia, MD), and the 11-day confinement study was conducted at a single research center (Celerion, Lincoln, NE) between April and June 2017 in accordance with the ethical standards in the Declaration of Helsinki and applicable sections of the United States Code of Federal Regulations (21 CFR Parts 50, 54, 56, and 312 Subpart D), and ICH E6 Good Clinical Practice guidelines. The results for Vuse Ciro and Vuse Vibe are reported in this manuscript with the same subjects and data for UB cigarettes and gum as in the previous publication^34^.

Potential participants were recruited via standard advertisements (print media, radio, and television) and through recruitment databases. Eligible participants were men and women aged 21–60 years in generally good health who self-reported smoking at least 10 filtered non-menthol 83–100 mm combustible cigarettes per day for 6 months or longer. The first cigarette of the day had to typically be smoked within 30 min of waking, and participants were not to have used an ENDS in the 30 days prior to screening. Smoking status was confirmed via levels of carbon monoxide in expired breath at screening (≥ 15 ppm). Smokers who expressed an interest in quitting within 30 days of screening, who were pregnant or breastfeeding, or who were using systemic estrogen-containing contraception or hormone-replacement therapies were excluded. Other key exclusion factors were hemoglobin levels lower than 12.5 g/dl (women) or 13.0 g/dl (men), a history of bleeding disorders, and a recent whole blood donation. All subjects underwent an informed consent process prior to any study procedures, and once enrolled, were informed that they could leave the study at any time.

### Study products

Vuse Vibe and Vuse Ciro products are pre-filled, closed-tank vapor products (RJR Vapor Company, Winston-Salem, North Carolina) that were commercially available at the time the study was conducted. The Vuse Vibe power unit is powered by a ≥ 550 mAh rechargeable battery and is paired with a cartridge containing 1.9 mL of e-liquid with 3% nicotine content by weight (36 mg/mL) and a propylene glycol to glycerin ratio (PG/VG) of 20/80. The Vuse Ciro power unit has a ≥ 260 mAh battery and uses a cartridge containing 0.9 mL of e-liquid with 1.5% nicotine by weight (17.7 mg/mL) and a 29/71 PG/VG ratio. Both Vuse Vibe and Vuse Ciro were used with Original, tobacco-flavored e-liquids.

Comparator products were participants’ UB cigarettes as a high abuse liability product and Nicorette® White Ice Mint nicotine polacrilex nicotine replacement therapy (NRT) gum containing 4 mg of nicotine (GlaxoSmithKline, USA) as a low abuse liability product.

### Study procedures

Within 45 days of the screening visit, eligible participants attended the study center for an 11-day confinement study that was divided into five different 48-h Study Periods (Supplementary Figure S1). After eligibility was reconfirmed at study check-in, participants were enrolled and randomized via a Williams design to one of ten potential product use sequences. Over the course of the confinement, participants sequentially used five study products (Vuse Vibe, Vuse Ciro, Vuse Solo, UB cigarette, and nicotine gum), one in each Study Period, according to their randomization schedule.

The 48-h Study Periods per product included one and a half days of product familiarization, in which subjects were required to participate in a minimum of six ad libitum use sessions of their assigned non-UB study products. The parameters for ad libitum use during product familiarization and AL test sessions were as follows: up to 10 min for the UB cigarettes, approximately 10 min (± 10 s) for the assigned ENDS, and 30 min (± 10 s) for the nicotine gum. During product familiarization, subjects were allowed to use the products longer if desired. On the last half-day of each Study Period, following a minimum 12-h overnight nicotine abstinence period, subjects participated in a 4-h test session using their assigned product. Baseline blood sampling and urge-to-smoke assessments were made prior to product use during the test session. During and after product use, participants underwent assessments for subjective effects and physiological changes and blood sampling for nicotine uptake. Additional details of study procedures are published in Campbell et al.^34^.

### Study assessments

Level of dependence on cigarettes at baseline was assessed with the Fagerström Test for Nicotine Dependence (FTND), in which a score of 0–2 indicates low dependence and 8–10 indicates high dependence^35^. Subjective effects were evaluated using responses to five different single-item questionnaires administered on paper forms using an 11-point numerical rating scale. In brief, the measures included product liking at a given time (“At this moment, how much do you like the product?”), product effects (“Rate the degree to which you feel positive/negative effects of the product at this moment.”), urge to smoke (“How strong is your current urge to smoke your usual brand cigarette?”), overall product liking (“Overall, how much do you like the product?”), and overall intent to use a product again (“Rate the degree to which you would like to use the product again.”). All responses were scored on rating scales of 0 to 10, where 10 represented “strongly like,” “extremely positive/negative effects,” “extremely strong urge,” “strong liking,” or “very much”, respectively; and 0 indicated “strongly dislike,” “strong disliking,” “no positive/negative effects,” “no urge,” “strongly dislike,” or “not at all.” The measurement of nicotine in (EDTA) plasma was performed by Celerion (Lincoln, NE) utilizing a methodology validated to meet FDA guidance. Blood pressure and heart rate were measured before and throughout the test session as previously described^34^. Timepoints for study assessments are in Supplementary Table S1.

Safety was assessed by monitoring adverse events (AEs), clinical laboratory tests, vital sign measurements, physical examinations, electrocardiography, and, in women, serum pregnancy and serum follicle-stimulating hormone tests. AEs were coded by organ class in accordance with the MedDRA Version 20.0. Severity was classified as mild, moderate, or severe.

### Outcomes

Pharmacokinetic outcomes related to nicotine uptake in plasma following use of the study products were calculated using baseline-adjusted nicotine levels. The specific outcome variables were area under the curve for nicotine concentration versus time from 0 to 15 and 240 min (AUC_0–15_ and AUC_0–240_), maximum concentration (C_max_), and time to maximum concentration (T_max_). Subjective measures included product liking (PL), calculated as the area under the effect curve (AUEC) for score versus time from 15 to 240 min after the start of use (AUEC_15–240_), maximum PL score (E_max_), and overall product liking (OPL) and overall intent to use again (OIUA) at 240 min after the start of use. Positive and negative experiences were calculated as AUEC_15–240_ and E_max_ for positive and negative experiences; and urge to smoke was calculated as AUEC for a score versus time from 0 to 15 min after the start of use (AUEC_0–15_), AUEC_0–240_, minimum score (E_min_), and time to minimum (T_min_). Lastly, pharmacodynamic measures assessed were maximum absolute physiological changes (systolic and diastolic blood pressure and heart rate) from before to after investigational product use were calculated.

### Statistical analysis

The sample size calculation was based on data from a first-generation ENDS study relevant to the primary endpoints for this study^28^. Using these data and considering requirements of the Williams design randomization method, it was calculated that 30 participants completing all test sessions would achieve 80% power to detect differences between ENDS and UB cigarettes at a two-sided significance level of *p* = 0.0013 (Bonferroni-adjusted for multiple comparisons). To allow for potential study dropouts, 40 participants were enrolled.

Data management and statistical analyses were performed by Celerion with SAS version 9.3 (SAS, Cary, NC). Phoenix® WinNonlin® version 6.3 or higher (Certara, LP, Princeton, NJ) was used to calculate non-compartmental nicotine pharmacokinetics and subjective responses.

All data were summarized by descriptive statistics, including arithmetic mean and standard deviation (SD) or median and range for continuous data and frequency counts for categorical data. For comparative analysis, Vuse Vibe and Vuse Ciro were compared with UB cigarettes and nicotine gum, and no comparisons were made between the Vuse products. All participants with at least one post-baseline investigational product assessment and evaluable data were included in the analyses. Evaluable data were defined as having first and last values to calculate changes over time (e.g., AUC_0–240_) and being within the expected range (e.g., T_max_ < 120 min).

Pharmacokinetic and subjective measures were analyzed with ANOVA mixed-effects models, except for urge to smoke, for which we used a mixed-effects ANCOVA model. In all models, sequence, period (i.e., order within assigned group of test sessions), and product were included as fixed effects, and participant-nested-within-sequence was included as a random effect. Pharmacokinetic AUC and C_max_ values were analyzed on the natural log scale, and T_max_ was analyzed on the original scale. For the analysis of nicotine uptake, concentrations in plasma were adjusted for baseline values with the following formula:

$$C^{\prime}_{t} = {\text{ C}}_{{\text{t}}} {-}{\text{C}}_{0} \left( {1/2} \right)^{{{\text{t}}/{\text{t}}1/2}}$$

where $C^{\prime}_{{\text{t}}}$ was the adjusted concentration at time t, C_t_ was the observed concentration at time t, C_0_ was the concentration at time 0, t was time in minutes, t_1/2_ was an average nicotine half-life of 120 min. Nicotine elimination was assumed to follow first-order kinetics. Baseline was the plasma nicotine concentration directly before investigational product use (− 0.5 [preferred] or − 5 min). The resulting baseline concentrations were subtracted from the observed concentrations in plasma at each subsequent timepoint (Supplementary Table S1). Any negative values after adjustment were set to zero. Measured nicotine concentrations in plasma that were below the lower limit of quantitation (0.2 ng/mL) were set to half this value for data summarization and statistical analysis. Any subjects with T_max_ ≥ 120 min were considered not to have used the study products effectively during the test session, and the subjective effects and PK data analyses were performed both with and without the potentially compromised test session data. The data presented herein excludes 5 subjects with exceptionally long T_max_ values (≥ 120 min). Statistical comparisons with all subjects are provided in the supplemental materials (Supplemental Table S4).

## Results

The original study included assessments of three ENDS products relative to UB cigarettes and nicotine gum. The results for one product, Vuse Solo G2, were previously published elsewhere^34^. This paper presents the results for the two remaining products, Vuse Ciro and Vuse Vibe.

### Study participants

A total of 93 candidates were screened, and 40 subjects were randomized. Those that were not randomized either failed inclusion/exclusion criteria at screening (n = 40) or were lost to follow-up (n = 13). Thirty-eight participants completed all test sessions for Vuse Ciro and NRT gum, and 39 participants completed all test sessions for Vuse Vibe and UB cigarettes. Two participants withdrew from the study, one due to a vasovagal reaction related to blood draws (withdrawn by the principal investigator) and one due to participant’s choice before receiving any study products.

The demographic and baseline characteristics of all randomized subjects were previously reported in Campbell et al.^34^, and are included in Supplementary Table 1. The study population was predominantly male (70%), white (95%), and non-Hispanic (95%). The mean (age was 41.2 (standard deviation [SD]) = 11.0) years. Mean individual cigarette use was 18 cigarettes per day (SD = 4.3), and the mean smoking duration was 20 years (SD = 12.7). All subjects were self-reported smokers of combustible cigarettes with limited ENDS experience. Dependence on cigarettes was moderate, with a mean score of 5.5, but the range of scores was 3.0–10.0, indicating notable variability among participants. No participant reported current regular use of ENDS before entering the study.

### Product use

The mean e-liquid weight difference before and after 10 min of ad libitum product use during the Vuse Vibe test sessions was 0.052 g ± 0.032 (range: 0.0032 g to 0.0457 g). For Vuse Ciro, the mean e-liquid weight used was 0.068 g ± 0.047 (range: 0.0124 g to 0.2000 g). These weights are equivalent to a mean nicotine aerosolization of 1.56 mg for Vuse Vibe and 1.02 mg for Vuse Ciro.

### Subjective measures

Product-liking scores at given times for Vuse Vibe and Vuse Ciro were consistently between those for usual brand cigarettes, which had the highest scores, and nicotine gum, which had the lowest scores (Table 1). All differences were statistically significant. Similarly, the scores for OIUA and positive effects were also intermediate between the two comparator products. The negative effects scores (AUEC_PEneg 15–240_ and E_max PEneg_) for Vuse Vibe and Vuse Ciro were not statistically significantly different from UB cigarettes. Compared to use of nicotine gum, the mean negative effects scores were lower, with all but AUEC_PEneg 15–240_ for Vuse Vibe being statistically significantly lower.

**Table 1:** Statistical comparisons of subjective measures parameters between Vuse Vibe or Vuse Ciro and the high- and low-AL comparators.

Parameter	UB cigarette	Nicotine gum	Vuse Vibe (3%)	Vuse Vibe *p* values		Vuse Ciro (1.5%)	Vuse Ciro * p* values	
	(N = 39)	(N = 36)	(N = 37)	P1	P2	(N = 36)	P3	P4
Overall Product Liking (OPL)	8.1	3.6	5.6^1,2^	< 0.0001	< 0.0001	6.2^1,2^	< 0.0001	< 0.0001
Product Liking (AUEC_PL 15–240_)*	1735.7	866.7	1230.8^1,2^	< 0.0001	< 0.0001	1363.5^1,2^	< 0.0001	< 0.0001
Product Liking (E_max PL_)*	8.8	5.0	6.6^1,2^	< 0.0001	< 0.0001	7.1^1,2^	< 0.0001	< 0.0001
Overall Intent To Use Again (OIUA)*	9.0	2.2	4.5^1,2^	< 0.0001	< 0.0001	5.3^1,2^	< 0.0001	< 0.0001
Positive Effects (AUEC_PE pos 15–240_)	925.7	578.4	753.2^1,2^	0.0131	0.0136	765.7^1,2^	0.0224	0.0088
Positive Effects (E_max PE pos_)	7.0	4.2	5.7^1,2^	0.0020	0.0009	5.8^1,2^	0.0047	0.0004
Negative Effects (AUEC _PE neg 15–240_)	341.4	499.5	390.0	0.4189	0.0751	327.7^2^	0.8209	0.0060
Negative Effects (E_max PE neg_)	2.9	4.3	3.1^2^	0.6204	0.0064	2.5^2^	0.3831	< 0.0001
Urge to Smoke (AUEC_UTS 0–15_)	70.6	110.4	97.3^1,2^	< 0.0001	0.0200	96.1^1,2^	< 0.0001	0.0120
Urge to Smoke (AUEC_UTS 0–240_)	1609.0	1861.8	1767.7^1^	0.0118	0.1408	1797.7^1^	0.0032	0.3205
Urge to Smoke (E_min UTS_)	2.7	5.8	5.0^1^	< 0.0001	0.0582	4.9^1,2^	< 0.0001	0.0368
Urge to Smoke (T_min UTS_, minutes)	14.7	33.0	16.8^2^	0.7429	0.0145	21.4	0.3007	0.0813

Least squares means from mixed-effect models are presented. 1 = statistically significant different from UB cigarette; 2 = statistically significant different from nicotine gum. The statistical significance thresholds were 0.0013 for the primary endpoints (*) and 0.05 for the secondary endpoints (all others). *p* values for statistical comparisons are indicated as follows: P1 = Vuse Vibe compared to UB cigarette; P2 = Vuse Vibe compared to nicotine gum; P3 = Vuse Ciro compared to UB cigarette; P4 = Vuse Ciro compared to nicotine gum. Note: The two ENDS products were not compared to each other, nor were the high- and low-AL comparators compared to each other. Abbreviations: AUEC_15–240_, area under the effect curve from 15 to 240 min after the start of product use; E_max_, maximum effect score; AUEC_0–15_, area under the effect curve from 0 to 15 min after the start of product use; AUEC_0–240_, area under the effect curve from 0 to 240 min after the start of product use; E_min UTS_, minimum effect score; T_min UTS_, time to minimum urge to smoke.

**Figure 1:**
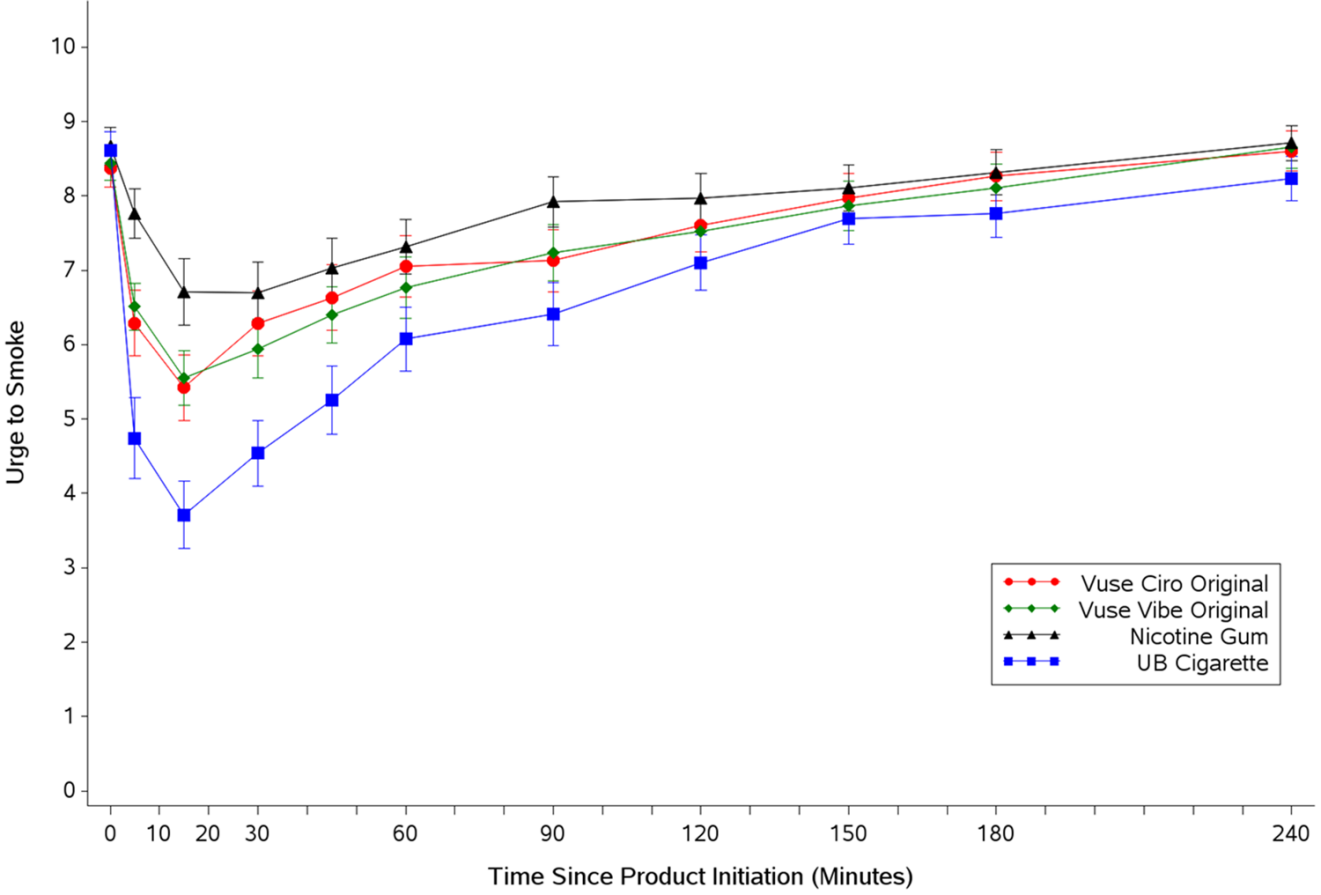
Arithmetic mean (SE) urge to smoke response profiles over four hours after initiation of product use. SE standard error, UB usual brand.

Vuse ENDS use suppressed the urge to smoke in the first fifteen minutes after initiation of product use to a greater degree than nicotine gum but to a lesser degree than UB cigarettes (Fig. 1). Over the entire 4-h test session, the mean UTS (AUEC_UTS 0–240_) for Vuse Vibe and Vuse Ciro was significantly higher than for UB cigarettes (*p* = 0.0118 and *p* = 0.0032, respectively). The AUEC_UTS 0–240_ values for Vuse Vibe and Vuse Ciro over the entire 240-min assessment were numerically lower than, but not statistically significantly different from nicotine gum. The minimum UTS value (T_min UTS_) was reached fastest, and the degree of effect was greatest with smoking (UB cigarette), followed by Vuse Vibe and Vuse Ciro, then nicotine gum. The minimum UTS score for Vuse Ciro was statistically significantly lower than both UB and nicotine gum. By 240 min, UTS values for all products had returned to near baseline (Fig. 1).

### Nicotine pharmacokinetics

**Figure 2:**
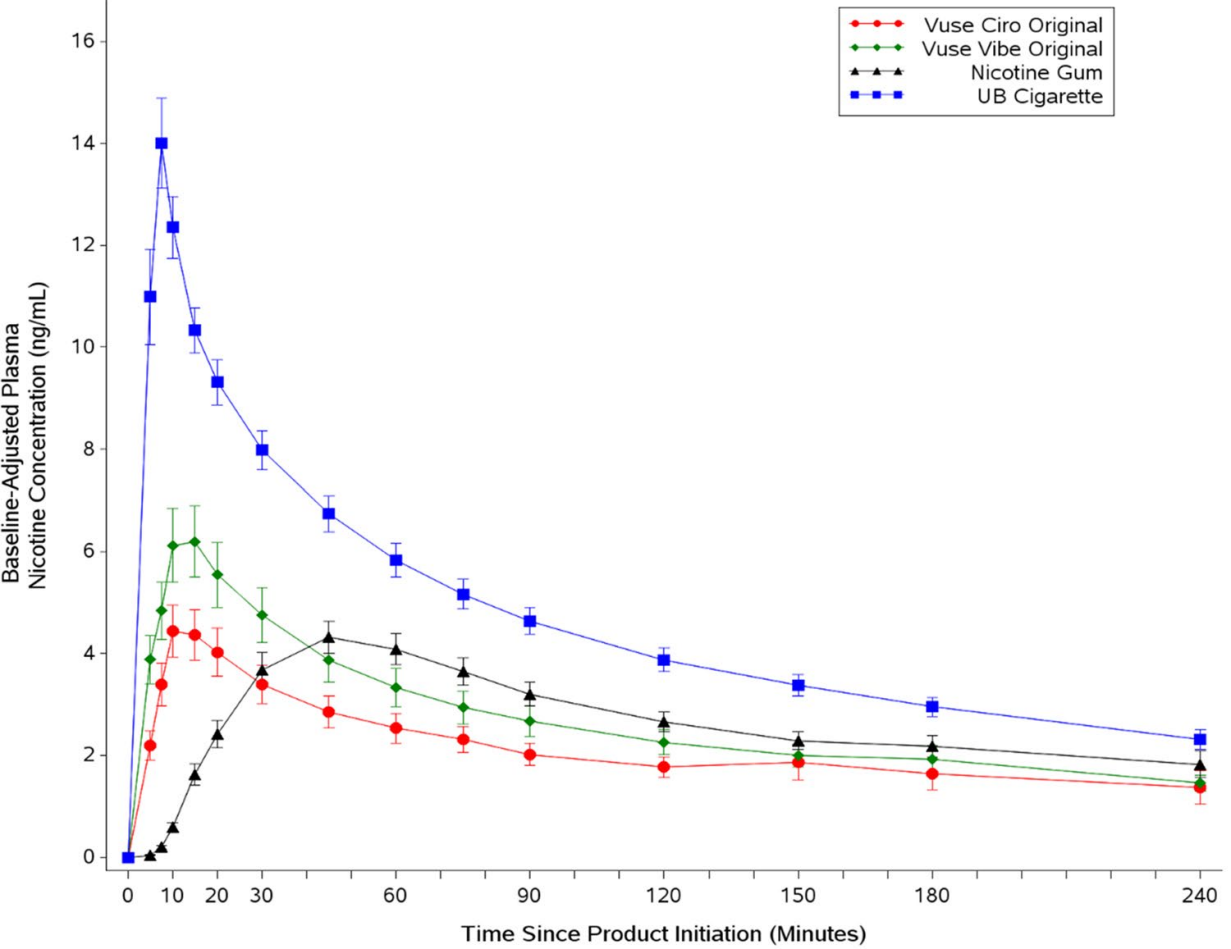
Arithmetic mean (SE) baseline-adjusted plasma nicotine concentration profiles. SE standard error, UB usual brand.

Nicotine concentrations in plasma were greatest for usual-brand cigarettes, followed by the two ENDS products and then nicotine gum throughout the 240 min after the start of the test sessions (Table 2). Compared with usual-brand cigarettes, the mean peak uptake (C_max_) and mean total uptake of nicotine (AUC_nic 0–240_) were statistically significantly lower (*p* < 0.001) with the ENDS products, regardless of nicotine level (ranging from 28 to 46% of UB cigarette for C_max_ and 36% to 48% of UB for AUC_nic 0–240_, depending on product (Fig. 2). When compared with nicotine gum, the mean peak uptakes for both ENDS products were not statistically significantly different. The mean total nicotine uptake for Vuse Ciro was statistically significantly lower than nicotine gum (*p* = 0.0011), whereas there was no statistically significant difference between the AUC_nic 0–240_ values for Vuse Vibe versus nicotine gum. The median T_max_ values for the Vuse products (~ 15 min) were statistically significantly longer than that of UB cigarette (7.6 min; *p* < 0.001) and statistically significantly shorter than that of nicotine gum (45 min; *p* < 0.001).

**Table 2:** Statistical comparisons of baseline-adjusted plasma nicotine uptake parameters between Vuse Vibe or Vuse Ciro and the high- and low-AL comparators.

PK Parameter^a^	UB cigarette	Nicotine gum	Vuse Vibe (3%)	Vuse Vibe * p* values		Vuse Ciro (1.5%)	Vuse Ciro * p* values	
	(N = 39)	(N = 36)	(N = 37)	P1	P2	(N = 36)	P3	P4
AUC_nic 0–15_ (ng*ng/mL)	140.7	4.67	50.15^1,2^	< 0.0001	< 0.0001	32.02^1,2^	< 0.0001	< 0.0001
AUC_nic 0–240_ (ng*ng/mL) *	1082	550.3	521.9^1^	< 0.0001	0.6037	390.1^1,2^	< 0.0001	0.0011
C_max_ (ng/mL)*	14.07	3.99	5.63^1^	< 0.0001	0.0049	3.87^1^	< 0.0001	0.7953
T_max_ (min)*	7.62	45.0	14.9^1,2^	< 0.0001	< 0.0001	15.0^1,2^	< 0.0001	< 0.0001

^a^Geometric least square means were used for the C_max_ and AUC statistical comparisons; median values were used for T_max_ statistical comparisons. Arithmetic means for C_max_ of Vuse Ciro, Vuse Vibe, UB, and nicotine gum were 4.99, 7.23, 15.02, and 4.55 ng/mL, respectively. 1 = statistically significant different from UB cigarette; 2 = statistically significant different from nicotine gum. The statistical significance thresholds were 0.0013 for the primary endpoints (*) and 0.05 for the secondary endpoints (all others). *p* values for statistical comparisons are indicated as follows: P1 = Vuse Vibe compared to UB cigarette; P2 = Vuse Vibe compared to nicotine gum; P3 = Vuse Ciro compared to UB cigarette; P4 = Vuse Ciro compared to nicotine gum. Note: The ENDS products were compared individually to UB cigarette and nicotine. No other comparisons were made. Abbreviations: C_max_, maximum concentration; AUC_nic 0–15_, area under the curve from 0 to 15 min after the start of product use; AUC_nic 0–240_, area under the curve from 0 to 240 min after the start of product use; T_max_, time to maximum concentration.

### Physiological effects

Mean systolic and diastolic blood pressure and heart rates at baseline were similar in all groups and were generally consistent throughout the test sessions (Table 3). The maximum increase in heart rate was lowest after use of Vuse Ciro (7.51 bpm), which was statistically significantly different from that of the highest, UB cigarette (12.2 bpm). The maximum increases after use of Vuse Vibe and nicotine gum were 11.47 and 9.08 bpm, respectively. No other comparisons were statistically significant.

**Table 3:** Mean (SD) of systolic blood pressure (mmHg), diastolic blood pressure (mmHg), and heart rate (bpm) with use of products.

Systolic blood pressure (mmHg)	Time since product use (min)							
	0	15	30	45	60	120	180	240
UB cigarette	110 (10.8)	111 (11.9)	110 (10.7)	109 (9.4)	109 (10.1)	108 (9.1)	109 (8.5)	112 (11.3)
Vuse Vibe	110 (7.9)	110 (9.8)	109 (9.1)	109 (8.6)	109 (8.2)	107 (8.7)	106 (7.0)	111 (9.9)
Vuse Ciro	111 (12.7)	111 (8.6)	110 (10.1)	109 (8.9)	111 (9.4)	108 (8.8)	111 (11.7)	113 (10.2)
Nicotine gum	110 (9.5)	109 (7.6)	110 (11.1)	108 (11.6)	107 (10.8)	110 (10.2)	108 (11.4)	111 (10.3)

Diastolic blood pressure (mmHg)	Time since product use (min)							
	0	15	30	45	60	120	180	240
UB cigarette	69 (6.1)	70 (6.7)	70 (6.3)	70 (5.4)	70 (7.1)	70 (6.3)	69 (5.7)	70 (6.0)
Vuse Vibe	69 (5.3)	71 (6.1)	69 (5.5)	70 (5.3)	69 (7.1)	68 (5.6)	68 (6.3)	71 (5.8)
Vuse Ciro	69 (6.9)	71 (5.4)	68 (6.5)	69 (7.0)	69 (6.0)	68 (6.6)	70 (7.5)	72 (7.0)
Nicotine gum	69 (5.7)	69 (5.9)	70 (7.4)	70 (7.9)	69 (8.2)	70 (6.9)	70 (6.3)	71 (6.8)

Heart rate (bpm)	Time since product use (min)							
	0	15	30	45	60	120	180	240
UB cigarette	68.4 (8.78)	80.1 (11.72)	73.0 (8.49)	69.6 (8.96)	69.5 (7.83)	64.5 (8.72)	63.1 (10.71)	62.9 (10.06)
Vuse Vibe	71.2 (9.23)	79.6 (9.89)	73.1 (10.8)	70.1 (9.45)	67.8 (8.66)	64.4 (7.96)	64.7 (8.50)	64.9 (9.93)
Vuse Ciro	70.3 (7.44)	72.8 (9.86)	69.4 (8.43)	70.5 (10.71)	66.7 (9.05)	63.7 (7.76)	62.6 (9.00)	64.8 (7.41)
Nicotine gum	70.9 (9.82)	74.3 (10.81)	75.0 (8.17)	72.2 (6.96)	71.2 (8.81)	64.9 (9.59)	64.6 (9.21)	63.9 (10.90)

### Safety

All study products were tolerated well, and no serious adverse events were reported. Sixty-seven adverse events were reported in 24 (60%) participants, with mild headache being the most common. Two headaches were judged to be related to use of Vuse Vibe, three related to nicotine gum use, and one related to smoking UB. All other adverse events were reported in three or fewer participants. Twenty-four AEs were judged to be likely related or related to investigational product use, with four related to Vuse Vibe, two related to Vuse Ciro, three related to UB cigarette, and fifteen related to nicotine gum (details provided in Supplementary Table S3). One participant was withdrawn from the study due to mild presyncope events related to blood draws during their first test session. All AEs resolved by the end of the study, except for four subjects with mild clinical laboratory AEs. Three of these were deemed unrelated to product use, and participants were instructed to follow up with a primary care physician. The fourth subject with a mild clinical laboratory AE was unable to be contacted after the study; therefore, the PI determined that a possibility still existed that there was a relationship to study product.

## Discussion

This study examined factors related to AL as well as likelihood of use for two tobacco-flavored ENDS products (Vuse Ciro [1.5% nicotine] and Vuse Vibe [3.0% nicotine]) in current smokers. Similar to previous studies of other ENDS, the results indicate that Vuse Ciro and Vuse Vibe have AL profiles below that of combustible cigarettes and either similar to or slightly higher than nicotine gum. After a single 10-min ad libitum use, a time frame selected to be consistent with previous AL studies^29^, smokers scored Vuse Ciro and Vuse Vibe lower than UB cigarettes but higher than nicotine gum for all product liking, positive effect, and intent to use again measures. Negative effects were scored lower than or similar to nicotine gum and on par with cigarettes. The timing of reductions in urge to smoke after use of Vuse Vibe and Vuse Ciro was more similar to UB cigarettes than to nicotine gum, and the urge to smoke scores over the first fifteen minutes after product use for the Vuse products were intermediate between the two comparators. Suppression of urges to smoke and reduction of aversive withdrawal symptoms are critical factors in migrating smokers away from cigarettes. Additional subjective effects results, such as product liking and positive and negative effects, support the conclusion that the abuse liability of Vuse Vibe and Vuse Ciro Original ENDS is significantly lower than that of combusted cigarettes and slightly greater than that of 4 mg NRT gum.

Nicotine uptake in the first 15 min after the start of product use was significantly higher and the median T_max_ was significantly shorter for the Vuse products than for nicotine gum, reflective of route of administration and absorption being generally faster for inhaled nicotine products compared to oral nicotine products. Early nicotine uptake likely contributed to the Vuse products more effectively suppressing urge to smoke than nicotine gum. In contrast, the median T_max_ values for the Vuse products were statistically significantly longer than that of UB cigarettes. The T_max_ differences for the Vuse products and UB cigarettes may be an artifact of study design, as the ENDS products were used for 10 min, and the time to completely smoke a cigarette is typically faster (approximately 5 to 7 min).

The maximum and overall nicotine uptake after using Vuse Vibe and Vuse Ciro was similar to after use of nicotine gum. Notably, overall nicotine uptake after use of Vuse Ciro was statistically significantly lower than after use of nicotine gum. Despite the similar (or lower) maximum and overall nicotine uptake for the ENDS products compared to nicotine gum, faster nicotine uptake and higher scores for product liking and intent to use again support the conclusion that Vuse Vibe and Vuse Ciro have a sufficient nicotine pharmacokinetic profile to support continued use and higher overall positive subjective effects profile supporting product use than nicotine gum. From a tobacco harm reduction perspective, this may increase the likelihood of product use and switching compared to nicotine gum in current smokers^15,36^. The overall results suggest that Vuse Ciro and Vuse Vibe are viable replacements for combustible cigarettes among smokers.

The FDA has acknowledged the criticality of information related to the nicotine pharmacological profiles of new tobacco and nicotine-containing products^37^. In their final rule outlining requirements for premarket tobacco product applications (PMTAs), the position of FDA on the need for AL data was clear. They stated that, “If FDA lacks sufficient information regarding the potential abuse liability of the new tobacco product, it intends to issue a marketing denial order for the new tobacco product.” In general, consideration of new tobacco products should balance the potential for increased exposure to existing harmful product constituents if products have high AL versus the potential for minimal use and adoption of the new product, if a product has low AL. The latter could lead to smokers’ continued use of the most harmful form of nicotine delivery—combusted cigarettes^38^. Due to further assessment and characterization of new nicotine-containing products, there is a growing body of literature in vapor products with both cross-category (i.e., NRTs or combusted cigarettes) and within-category (i.e., ENDS products) comparisons. AL testing results for Vuse Solo G1 and G2, RELX Infinity, myBlu, JUUL, eGO, and BIDI Stick ENDS products have been published in recent years, and the results and conclusions for all products are generally consistent with AL being lower than cigarettes and higher than NRT^23–29^. It should be noted that the results from different studies cannot be directly compared because of differences in study design, including puffing protocols and subjects’ past experience with ENDS^39,40^. Additionally, nicotine uptake parameters are generally correlated with e-liquid nicotine content, but the relationship is not consistent across products. Other factors that affect puffing behaviors will also affect PK parameters. For example, the flavor profile, the ratio of propylene glycol to glycerin and aerosol characteristics (e.g., mouth feel), product perceptions, and individual subjective experiences in using the product could all impact product use and PK profiles^15,41^. Notably, higher nicotine content levels in ENDS e-liquids do not necessarily lead to increased nicotine uptake in ad libitum use as high nicotine levels in inhaled aerosol can also be aversive, causing irritation or coughing and in turn reducing puffing intensity and product liking^42^. Our results with Vuse Ciro, a product with low e-liquid nicotine content but significantly higher positive subjective effects scores than nicotine gum, demonstrates that nicotine content is not the only factor that drives AL and product with lower nicotine levels can have significantly higher positive subjective effects.

The study had several strengths as well as limitations. Among its strengths, the study design included product acclimation sessions for familiarization with use of the product prior to the test sessions in which the PK, subjective measures, and physiological effects endpoints were assessed. The short 10-min test session use periods after compulsory nicotine abstention periods and the ad libitum puffing patterns more accurately represent ‘real-life’ nicotine uptake than would prescribed usage or initial product use without any prior experience^23^. The crossover design in which each subject provided endpoint data for the ENDS products, as well as the high- and low-AL comparative products, helped minimize between-subject variability of subjective responses and product use behaviors^43^. On the other hand, the study design does not provide information on future nicotine uptake following extended use of a product. However, based on extant literature, it is expected that nicotine uptake will increase somewhat with product use experience, further supporting the potential for these products to replace cigarettes among current smokers^44–46^.

The evidence demonstrated highest positive subjective effects measures scores for cigarettes, followed by the Vuse ENDS, and nicotine gum with the lowest scores. All nicotine uptake measures for the Vuse ENDS products were lower than that of UB cigarette, including peak nicotine uptake and overall nicotine uptake. These same measures for Vuse ENDS were either similar to or lower than nicotine gum, and the time course of nicotine uptake after use of the ENDS was more similar to that of combustible cigarettes than nicotine gum. In conclusion, the results indicate that the AL of each Vuse ENDS product tested is substantially lower than that of UB cigarettes and similar to that of nicotine gum.

### Supplementary Information

Supplementary Information.

## Data Availability

The applicable data generated or analyzed during this study are included in this manuscript (and its supplementary tables). Additional datasets generated and/or analyzed during the study are available from the corresponding author upon reasonable request.
