# Supplementary material for: Abuse liability of two electronic nicotine delivery systems compared with combustible cigarettes and nicotine gum from an open-label randomized crossover study
Source: Sci Rep. 2023 Nov 2;13:18951. doi: 10.1038/s41598-023-45894-7 (PMC10622442; doi:10.1038/s41598-023-45894-7)
Supplement: Supplementary file 1 — Supplementary Information. [file 41598_2023_45894_MOESM1_ESM.docx]

# Supplementary Materials for “Abuse liability of two electronic nicotine delivery systems compared with combustible cigarettes and nicotine gum from an open-label randomized crossover study.”

Chris Campbell, Tao Jin, Elaine K. Round, Paul R. Nelson, and Sarah Baxter

## Supplementary Figure S1. Study Schematic


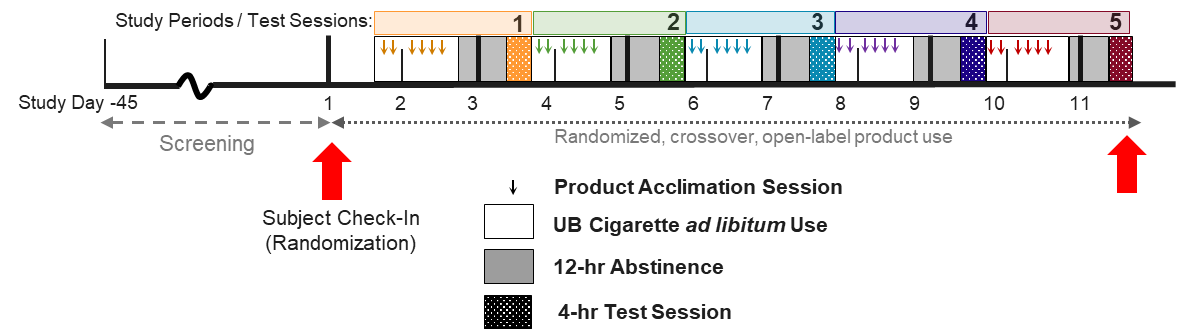


*This figure was adapted from the one previously published in Campbell et al., 2022.

## Supplemental Table S1: Time points for data collection

| **Study Assessments** | **Timepoints** |
| --- | --- |
| **Urge to Smoke** | -5, 5, 15, 30, 45, 60, 90, 120, 150, 180, and 240 minutes |
| **Product Liking / Product Effects** | 15, 30, 45, 60, 90, 120, 150, 180, and 240 minutes |
| **Overall Product Liking / Intent to Use Again** | 240 minutes |
| **Blood Draws for PK** | -5, -0.5, 5, 7.5, 10, 15, 20, 30, 45, 60, 75, 90, 120, 150, 180, and 240 minutes. |
| **Blood Pressure / Heart Rate** | -5, 15, 30, 45, 60, 120, 180, and 240 minutes |

## Supplementary Table S2. Demographic and baseline characteristics

| Parameter | All Enrolled Subjects (n=40) |
| --- | --- |
| Age (years) | 41.2 (±11.01) |
| Weight (kg) | 84.1 (±17.44) |
| Height (cm) | 172.9 (±8.74) |
| BMI (kg/m^2^) | 28.07 (±5.040) |
| Sex |  |
| Male | 28 (70%) |
| Female | 12 (30%) |
| Ethnicity |  |
| Hispanic/Latino | 2 (5%) |
| Not Hispanic/Latino | 38 (95%) |
| Race |  |
| White | 38 (95%) |
| Black/African American | 1 (3%) |
| Native Hawaiian/Other Pacific Islander | 0 (0%) |
| Asian | 0 (0%) |
| American Indian/Alaska Native | 1 (3%) |
| Multiple | 0 (0%) |
| Smoking |  |
| Number of years smoked | 19.7 (±12.7) |
| Number of cigarettes smoked per day | 18.1 (±4.26) |
| Level of dependence on nicotine (FTND) | 5.5 (±1.54) |

Data are mean (±SD) or number (%). Abbreviations: BMI = body-mass index, FTND = Fagerström Test for Nicotine Dependence

## Supplementary Table S3. Summary of adverse events

| **Product (total subjects; number of subjects reporting AEs / number of episodes)** | **Total Number of AEs Judged as ‘Not Related’ or ‘Unlikely Related’^a^** | **Total Number of AEs Judged as ‘Related’ or ‘Possibly Related’^b^** |
| --- | --- | --- |
| **Usual Brand Cigarette (N=39; 7 / 12)** | **9** | **3** |
| Contusion | 1 | 0 |
| Dizziness | 0 | 1 |
| Headache | 0 | 1 |
| Hyperhidrosis | 1 | 0 |
| Lymphadenopathy | 1 | 0 |
| Nausea | 0 | 1 |
| Pain in extremity | 1 | 0 |
| Presyncope | 1 | 0 |
| Vessel puncture site pain | 2 | 0 |
| Vessel puncture site reaction | 1 | 0 |
| Vessel puncture site swelling | 1 | 0 |
| **Vuse Vibe (N=39; 8 / 14)** | **10** | **4** |
| Flatulence | 1 | 0 |
| Headache | 3 | 2 |
| Nasal congestion | 2 | 0 |
| Nausea | 1 | 0 |
| Oropharyngeal pain | 0 | 1 |
| Procedural dizziness | 1 | 0 |
| Productive cough | 0 | 1 |
| Sneezing | 1 | 0 |
| Vessel puncture site pain | 1 | 0 |
| **Vuse Ciro (N=38; 4 / 7)** | **5** | **2** |
| Dry mouth | 0 | 1 |
| Dysmenorrhoea | 1 | 0 |
| Dyspepsia | 0 | 1 |
| Dysuria | 1 | 0 |
| Micturition urgency | 1 | 0 |
| Nasal congestion | 1 | 0 |
| Skin abrasion | 1 | 0 |
| **Nicotine Gum (5 / 34)** | **19** | **15** |
| Abdominal distension | 0 | 1 |
| Arthralgia | 1 | 0 |
| Back pain | 2 | 0 |
| Blood urine present^c^ | 1 | 0 |
| Burning sensation | 1 | 0 |
| Constipation | 3 | 0 |
| Diarrhoea | 1 | 0 |
| Dizziness | 0 | 3 |
| Dyspepsia | 0 | 1 |
| Erythema | 1 | 0 |
| Feeling hot | 0 | 1 |
| Headache | 0 | 3 |
| Hiccups | 0 | 1 |
| Lymphocyte count increased^c^ | 2 | 1 |
| Nausea | 0 | 2 |
| Oral discomfort | 0 | 1 |
| Paraesthesia | 1 | 0 |
| Peripheral coldness | 1 | 0 |
| Presyncope^d^ | 2 | 0 |
| Throat irritation | 0 | 1 |
| Vessel puncture site pain | 2 | 0 |
| White blood cell count increased^c^ | 1 | 0 |
| During each 48-hour study period, subjects used the Study IP to which they were randomly assigned during Product Acclimation Sessions and a test session. All AEs were assigned to the randomized Study IP and relatedness to use of that study product was assessed by the Principal Investigator (PI) responsible for study conduct.  ^a^ AEs that were judged as ‘not related’ or ‘unlikely related’ were those that did not follow a reasonable temporal sequence from use of the IP or could be reasonably explained by other factors (i.e., underlying disease, complications, or concomitant drugs).  ^b^ AEs that were judged as ‘related’ or ‘possibly related’ were those that followed a reasonable temporal sequence from use of the IP (including the course after withdrawal of the IP) and that can be excluded as being possibly caused by other factors.  ^c^ Denotes mild AEs that were identified in four subjects during end-of-study clinical laboratory assessments. Three of these were deemed unrelated to product use and participants were instructed to follow-up with a primary care physician. The fourth subject with a mild clinical laboratory AE was unable to be contacted after the study; therefore, the PI determined that a possibility still existed that there was a relationship to study product.  ^d^ These two presyncope events (i.e., vasovagal reactions related to blood draws) occurred in the same subject during Day 1 (one in the morning, one in the afternoon) of the first study period. The subject was discontinued from the study by the PI. | | |

## Supplementary Table S4: Statistical comparisons of between Vuse Vibe and the high- and low-AL comparators and between Vuse Ciro and the high- and low-AL comparators (All subjects population)

| **Parameter ^*^** | **UB cigarette** | **Nicotine gum** | **Vuse Vibe (3%)** | **Vuse Ciro (1.5%)** |  |
| --- | --- | --- | --- | --- | --- |
|  | **(N=38)** | **(N=38)** | **(N=38)** | **(N=38)** |  |
| Overall Product Liking (OPL) | 8.14 | 3.58 | 5.59^1,2^ | 6.22^1,2^ |  |
| Product Liking (AUEC_PL_ _15-240_)* | 1735.99 | 890.52 | 1210.29^1,2^ | 1334.23^1,2^ |  |
| Product Liking (E_max PL_)* | 8.83 | 5.15 | 6.52^1,2^ | 6.92^1,2^ |  |
| Overall Intent to Use Again (OIUA)* | 8.98 | 2.24 | 4.48^1,2^ | 5.30^1,2^ |  |
| Positive Effects (AUEC_PE pos_ _15-240_) | 926.02 | 579.66 | 746.46^1,2^ | 752.58^1,2^ |  |
| Positive Effects (E_max PE pos_) | 7.02 | 4.34 | 5.58^1,2^ | 5.76^1,2^ |  |
| Negative Effects (AUEC _PE neg_ _15-240_) | 341.64 | 506.53 | 389.36^2^ | 312.00^2^ |  |
| Negative Effects (E_max PE neg_) | 2.91 | 4.27 | 3.13^2^ | 2.47^2^ |  |
| Urge to Smoke (AUEC_UTS 0-15_) | 70.71 | 110.29 | 98.22^1,2^ | 95.98^1,2^ |  |
| Urge to Smoke (AUEC_UTS 0-240_) | 1609.86 | 1856.71 | 1780.81^1^ | 1809.82^1^ |  |
| Urge to Smoke (E_min UTS_) | 2.68 | 5.86 | 5.04^1^ | 4.88^1,2^ |  |
| Urge to Smoke (T_min UTS_, minutes) | 14.67 | 32.00 | 16.75^2^ | 21.12 |  |
| AUC_nic 0-15_ (ng*ng/mL) | 140.8 | 4.605 | 47.30^1,2^ | 31.81^1,2^ |  |
| AUC_nic 0-240_ (ng*ng/mL) * | 1082 | 558.8 | 511.6^1^ | 398.7^1^ |  |
| C_max_ (ng/mL)* | 14.06 | 4.11 | 5.49^1^ | 3.888^1,2^ |  |
| T_max_ (min)* | 7.62 | 45.03 | 14.93^1,2^ | 14.98^1,2^ |  |
| ^*^ Least squares means from mixed-effect models are presented for subjective effects; geometric least squares means are presented for PK parameters  1= statistically significant different from UB cigarette; 2 = statistically significant different from nicotine gum. The statistical significance thresholds were 0.0013 for the primary endpoints (*) and 0.05 for the secondary endpoints (all others). Note: The two ENDS products were not compared to each other, nor were the high- and low-AL comparators compared to each other.  Abbreviations: AUEC_15–240_, area under the effect curve from 15 to 240 minutes after the start of product use; E_max_, maximum effect score; AUEC_0–15_, area under the effect curve from 0 to 15 minutes after the start of product use; AUEC_0–240_, area under the effect curve from 0 to 240 minutes after the start of product use; E_min_, minimum effect score; T_min_, time to minimum urge to smoke. | | | | | |
